# 影像引导射频消融治疗肺部肿瘤专家共识（2018年版）

**DOI:** 10.3779/j.issn.1009-3419.2018.02.09

**Published:** 2018-02-20

**Authors:** 宝东 刘, 欣 叶, 卫君 范, 晓光 李, 威建 冯, 强 卢, 宇 毛, 征宇 林, 鲁 李, 一平 庄, 旭东 倪, 加林 沈, 毅立 傅, 建军 韩, 忱瑞 李, 晨 柳, 武威 杨, 志勇 苏, 志远 吴, 磊 刘

**Affiliations:** 1 100053 北京, 首都医科大学宣武医院胸外科 Department of Thoracic Surgery, Xuanwu Hospital, Capital Medical University, Beijing 100053, China; 2 250100 济南, 山东大学附属省立医院肿瘤科 Department of Oncolog y, Shandong Provincial Hospital Affiliated to Shandong University, Jinan 250100, China; 3 510060 广州, 中山大学肿瘤医院影像与微创介入中心 Imaging and Interventional Center, Sun Yat-sen University Cancer Center, Guangzhou 510060, China; 4 100005 北京, 北京医院肿瘤微创中心 Department of Tumor Minimally Invasive Therapy, Beijing Hospital, Beijing 100005, China; 5 100038 北京, 首都医科大学附属复兴医院肿瘤科 Department of Oncolog y, Fuxing Hospital, Capital Medical University, Beijing 100038, China; 6 710038 西安, 空军军医大学唐都医院胸外科 Department of Thoracic Surgery, Tangdu Hospital, Fourth Military Medical University, Xi'an 710038, China; 7 010020 呼和浩特, 内蒙古自治区呼和浩特市第一医院胸外科 Department of Thoracic Surgery, Hohhot First Hospital, Inner Mongolia, Hohhot 010020, China; 8 350005 福州, 福建医科大学附属第一医院介入科 Department of Interventional Therapy, the First Affiliated Hospital of Fujian Medical University, Fuzhou 350005, China; 9 100101 北京, 解放军306医院胸外科 Department of Thoracic Surgery, 306th Hospital of PLA, Beijing 100101, China; 10 210009 南京, 江苏省肿瘤医院介入科 Department of Interventional Therapy, Jiangsu Cancer Hospital, Nanjing 210009, China; 11 200001 上海, 复旦大学附属中山医院胸外科 Department of Thoracic Surgery, Zhongshan Hospital, Fudan University, Shanghai 200001, China; 12 200001 上海, 上海交通大学医学院附属仁济医院南院肿瘤介入科 Department of Tumor Interventional Therapy, Renji Hospital, Shanghai Jiaotong University School of Medicine, Shanghai 200001, China; 13 100020 北京, 首都医科大学附属北京朝阳医院胸外科 Department of Thoracic Surgery, Chaoyang Hospital, Capital medical University, Beijing 100020, China; 14 250117 济南, 山东省肿瘤医院微创介入科 Department of Minimally Invasive Interventional Therapy, Shandong Provincial Tumor Hospital, Jinan 250117, China; 15 100020 北京, 中国医学科学院肿瘤医院介入治疗科 Department of Interventional Therapy, Cancer Hospital, Chinese Academy of Medical Sciences, Beijing 100020, China; 16 100142 北京, 北京大学肿瘤医院介入治疗科 Department of Interventional Therapy, Beijing Cancer Hospital, Beijing 100142, China; 17 100071 北京, 解放军307医院肿瘤微创治疗科 Department of Tumor Minimally Invasive Therapy, 307th Hospital of PLA, Beijing 100071, China; 18 024005 赤峰, 赤峰学院附属医院胸外科 Affiliated Hospital of Chifeng University, Chifeng 024005, China; 19 200025 上海, 上海交通大学医学院附属瑞金医院放射介入科 Department of Interventional Radiolog y, Ruijin Hospital, Shanghai Jiaotong University School of Medicine, Shanghai 200025, China

## 1 概述

肺癌是最常见的恶性肿瘤之一，在世界各地，肺癌均居恶性肿瘤死亡构成比的第一位，其发病率和死亡率仍在不断升高。据世界卫生组织（World Health Organization, WHO）下属的国际癌症研究机构（International Agency for Research on Cancer, IARC）出版的GLOBOCAN 2012估计：全世界肺癌新发病例182.5万（男性124.2万，女性58.3万）；肺癌死亡病例159.0万（男性109.9万，居首位；女性49.1万，居第2位）^[1]^。与30年前相比，我国肺癌死亡率上升了465%，每年大约有60万人死于肺癌^[2, 3]^。根据全国肿瘤登记中心估计，2012年肺癌新发73.3万人（男性50.9万，居首位；女性22.4万，居第2位）；肺癌死亡61.0万人（男性43.2万，女性17.8万，男女均居首位）；中国约占世界新发肺癌的35.8%，世界肺癌死亡的37.6%，5年生存率16.1%^[2]^。

肺癌多发生于中老年人，临床上有1/4-1/3的肺癌患者存在合并症，手术存在一定风险^[3, 4]^（表 1）。根据手术风险划分为低风险组（good-risk）、高风险组（high-risk）和不能手术组（medically inoperable）。针对低风险组患者，肺叶切除和淋巴结清扫仍然是早期肺癌的标准术式；高风险组患者可以行亚肺叶切除术（包括肺段切除和楔形切除）；对不能手术组可以采用肿瘤热消融（tumor thermal ablation）和立体定向放射治疗（stereotactic body radiation therapy, SBRT）等。肿瘤热消融是指利用热产生的生物学效应直接导致靶肿瘤发生凝固性坏死（coagulation necrosis）的原位灭活技术。它具有微创、恢复快，安全、并发症少，适形、效果可靠，可重复、费用低等优点，被用于因肿瘤本身的原因，或者心肺功能不适合于外科手术治疗的肺癌病人。目前，国内外常用的肺部肿瘤热消融手段包括射频消融（radiofrequency ablation, RFA）、微波消融（microwave ablation, MWA）、冷冻消融（cryoablation）和激光消融（laser ablation）等。《中国原发性肺癌诊疗规范（2011年版）（2015年版）》（卫办医政发[2011]22号）推荐射频消融可以用于不能耐受手术早期肺癌患者的治疗^[4]^。在我国属于限制性医疗技术[《限制临床应用的医疗技术（2015版）》]。

**1:** 肺癌肺叶切除术排除标准 Exclusion criteria for lobectomy for primary lung cancer

排除标准
主要标准（至少1项） FEV_1_占预计值≤50% DLCO占预计值≤50%
次要标准（至少2项） 年龄≥75岁 FEV_1_占预计值51%-60% DLCO占预计值51%-60% 心脏超声或右心导管估计肺动脉压 > 40 mmHg 左心室射血分数≤40% 休息或运动时PO_2_≤55 mmHg或SPO_2_≤88% PCO_2_ > 45 mmHg
FEV_1_：第一秒用力肺活量；DLCO：一氧化碳弥散量；PO_2_：动脉血氧分压；SPO_2_：末梢血氧饱和度；PCO_2_：动脉血二氧化碳分压

射频消融的原理是应用频率 < 30 mHz（通常在460 kHz-480 kHz之间）的交变高频电流使肿瘤组织内离子发生高速震荡，互相摩擦，将射频能转化为热能，局部温度达到60 ℃-100 ℃时，肿瘤细胞发生凝固性坏死。凝固性坏死程度有赖于达到的温度和持续时间，影响因素包括热量传导与循环血液及细胞外液间的热对流^[5]^。2000年Dupuy等^[6]^报道3例经皮射频消融治疗肺部肿瘤患者，同年程庆书等^[7]^于国内首次报道了计算机断层扫描（computed tomography, CT）引导下锚状电极高温射频消融治疗肺部肿瘤105例患者的经验，拉开了射频消融应用于肺癌临床的序幕^[8-15]^。但是由于肺存在自主呼吸运动；肺属于含气器官、同时肺组织血运丰富，存在热沉降效应（heat sink effect）和阻抗高[阻抗平均（509±197）Ω]等特点；含气肺组织包绕肿瘤，存在烤箱效应（oven effect）；消融后肿瘤周围存在磨玻璃样阴影（ground-glass opacity, GGO）改变，与肿瘤实际凝固性坏死区不一致。导致了肺部肿瘤射频消融具有穿刺定位困难、局部进展率高^[16-18]^、疗效评价特殊和操作并发症多等特点。2014年10月，刘宝东教授和支修益教授组织国内有关肺部肿瘤射频消融领域的11位专家在北京，讨论达成了《影像引导射频消融治疗肺部肿瘤专家共识（2015年版）》，并在《中国肺癌杂志》上发表^[19]^，同时刊登在《中国胸心血管外科临床杂志》、《临床与病理杂志》、《*Journal of Thoracic Disease*》、《*Annals of Translational Medicine*》、《*Translational Lung Cancer Research*》和《*Chinese Clinical Oncology*》上^[20-25]^。共识发表 3年来，对我国肺部肿瘤射频消融的发展起到了积极的推动作用，但是也发现有需要进一步完善之处。为此，2017年12月3日，由《中国医疗保健国际交流促进会肺癌预防与控制分会》组织多位相关学科专家对共识进行了修订，旨在进一步规范肺部肿瘤射频消融的操作技术和疗效评估，为减少相关并发症的发生和提高治疗效果做出努力。

**3:** NSCLC的TNM分期标准 Stage criteria in the TNM staging system for NSCLC

TNM分期	
原发肿瘤（T）	
T_X_	原发肿瘤无法评估，或虽然痰或气管灌洗液中发现恶性细胞，但影像学或支气管镜检查未发现肿瘤
T0	无原发肿瘤证据
Tis	原位癌
T1	肿瘤最大径≤3 cm，由肺组织或脏层胸膜包绕，支气管镜下未见病变侵及叶支气管。侵犯主支气管，但未侵及隆突（浅表弥漫型，不论大小）。
T1a	肿瘤最大径≤1 cm
T1b	肿瘤最大径 > 1 cm，但≤2 cm
T1c	肿瘤最大径 > 2 cm，但≤3 cm
T2	肿瘤最大径 > 3 cm但≤5 cm，或具有以下任一特征（具有这些特征的T2肿瘤如果≤5 cm则归为T2）：累及主支气管，但距隆突 < 2 cm，但不侵犯隆突；或伴随全肺不张或阻塞性肺炎。
T2a	肿瘤最大径 > 3 cm，但≤4 cm
T2b	肿瘤最大径 > 4 cm，但≤5 cm
T3	肿瘤最大径 > 5 cm但≤7 cm，或直接侵及以下部位：胸壁（包括肺上沟瘤）、膈神经、壁层心包；或在同一肺叶内有其他转移结节
T4	肿瘤最大径 > 7 cm，或任何大小肿瘤侵及到以下部位：纵隔、膈肌、心脏、大血管、气管、食管、喉返神经、椎体、隆突；或在同侧不同肺叶内有其他转移结节
区域淋巴结（N）	
N_X_	区域淋巴结无法评估
N0	无区域淋巴结转移
N1	同侧气管周围和/或同侧肺门淋巴结及肺内淋巴结转移，包括原发病灶直接侵及所致
N2	同侧纵隔淋巴结和/或隆突下淋巴结转移
N3	对侧纵隔，对侧肺门，同侧或对侧斜角肌，或锁骨上淋巴结转移
远处转移（M）	
M_X_	远处转移不能评估
M0	无远处转移
M1	有远处转移
M1a	对侧肺叶内有转移结节；伴有胸膜结节或出现恶性胸膜或心包积液
M1b	远处器官单发转移灶
M1c	多个或单个器官多处转移
TNM：肿瘤-淋巴结-转移（tumor-node-metastasis）；NSCLC：非小细胞肺癌（non-small cell lung cancer）.	

## 2 操作平台

### 2.1 CT

是目前最常用和最准确的操作平台。CT密度分辨率高，能显示病灶横断面位置，清楚显示心脏、大血管与病灶的关系，可以避免损伤到心脏大血管、气管、食管等重要结构。它具有定位精确、及时发现并发症和评估疗效的优点。但是不能实时监测穿刺过程，只能提供静态的横截面图像，需要反复扫描。

### 2.2 B超

B超引导可实时监测，操作时间短，但它显示的病灶和穿刺位置没有CT那样直观清楚，只用于超声能观察到肿瘤全貌的靠近胸壁或与胸壁粘连的肿瘤。

### 2.3 其他新技术

如磁共振、C臂CT技术^[26, 27]^、正电子发射计算机断层扫描-计算机断层扫描（positron emission tomography-computed tomography, PET-CT）^[28]^、CT-纤维支气管镜^[29, 30]^、磁导航支气管镜（electromagnetic navigation bronchoscopy, ENB）^[31]^。

## 3 射频电极

### 3.1 单极射频电极

有1个活性电极，同时拥有1个或几个回路电极板。包括多针伸展型、冷循环型和灌注型等不同的设计。

#### 3.1.1 多针伸展型射频电极

是由弹性良好的多个电极针置于14 G-19 G套管针内制成的同轴电极，导入肿瘤组织后，通过针柄上的推进装置，将电极针推出针管，展开阵列排成，从而扩大了凝固坏死区。

#### 3.1.2 冷循环型射频电极

采用中空双腔设计，通过电动压力泵循环冷却水至针尖，对活性电极针进行冷却，防止针尖附近组织干燥和炭化，从而降低阻抗，产生更大、更有效的凝固坏死区。冷循环型射频电极可分为单束型及三针集束型，后者较前者单点消融体积更大。

#### 3.1.3 灌注型射频电极

射频电极的尖端有小孔，可通过小孔向消融组织内注射液体（常为生理盐水），提高组织电导性和热传导性，增大凝固坏死区，防止组织炭化^[32, 33]^。

### 3.2 双极射频电极

由2根电极针组成（分别为活性电极和回路电极），或在1根电极针的尖端同时具有活性电极和回路电极，无需回路电极板。体内有金属植入物及心脏起搏器的患者宜选用双极射频电极。

三种类型的射频电极在肺部肿瘤的射频消融中都可以使用，考虑到患者存在自主呼吸，肺活动度较大，建议选择多针伸展型电极针以便出针后覆盖肿瘤，减少射频电极移动对肺等正常组织的副损伤。而对邻近心脏大血管或气管支气管等重要结构的肿瘤消融时选择与之平行的单针（非多针伸展型）比较安全。

## 4 适应证和禁忌证

### 4.1 适应证

#### 4.1.1 治愈性消融（curative ablation）

是指通过射频消融能够使肺部肿瘤组织完全坏死，并有可能达到治愈和延长生存的目的。

##### 4.1.1.1 原发性肺癌^[34]^

Ⅰ期周围型早期非小细胞肺癌（non-small cell lung cancer, NSCLC）（肿瘤最大径≤3 cm，无淋巴结转移及远处转移），合并心肺功能差、高龄或拒绝手术的患者。包括多原发肺癌（multiple primary lung cancers, MPLC）。

##### 4.1.1.2 肺转移瘤^[35]^

原发灶得到有效控制者，同时单侧肺部转移瘤总数≤3个，双侧肺转移瘤总数≤5个，肿瘤最大径≤3 cm。

#### 4.1.2 姑息性消融（palliative ablation）

是指通过射频消融，最大限度地诱导肿瘤凝固性坏死，达到减轻肿瘤负荷、缓解症状和改善患者生活质量的目的。

##### 4.1.2.1

原发性肺癌^[15, 36-42]^肿瘤最大径 > 3 cm，进行多点或多次治疗，或联合其他治疗方法。

###### 4.1.2.1.1

原发性肺癌术后肺内孤立性复发。

###### 4.1.2.1.2

周围型NSCLC放化疗或分子靶向药物治疗后肺部肿瘤进展或者复发。

###### 4.1.2.1.3

周围型小细胞肺癌经过放化疗以后肿瘤进展或者复发。

###### 4.1.2.1.4

合并恶性胸腔积液的周围型肺癌在胸膜活检固定术后。

###### 4.1.2.1.5

肿瘤侵犯肋骨或胸椎椎体引起的难治性疼痛，对肿瘤局部骨侵犯处进行消融，可达到止痛效果。

##### 4.1.2.2 肺转移瘤

数量和大小超过治愈性消融标准者。

### 4.2 禁忌证

#### 4.2.1 绝对禁忌证

有严重出血倾向、血小板 < 50×10^9^/L和不能纠正的凝血功能障碍者（凝血酶原时间 > 18 s，凝血酶原活动度 < 40%）。抗凝治疗和/或抗血小板药物在消融前停用未超过5 d-7 d。

#### 4.2.2 相对禁忌证

##### 4.2.2.1

有广泛肺外转移者，预期生存 < 3个月。

##### 4.2.2.2

有严重合并症、感染期、免疫功能低下、肾功能不全者。

##### 4.2.2.3

心脏起搏器植入、金属物植入者。

##### 4.2.2.4

美国东部肿瘤协作组（Eastern Collaborative Oncology Group, ECOG）体力状态评分 > 3分（表 2）。

**2:** ECOG/Zubrod体力状态评分标准 ECOG/Zubrod performance status scale

活动状态	描述
0	无症状，完全主动活动，能够进行无限制的患病前活动
1	有症状，完全能行走，但重体力活动受限
2	有症状，能行走，生活可自理，清醒时间即白天卧床时间 < 50%
3	有症状，部分生活自理，清醒时间卧床>50%，但尚未卧床不起
4	完全失去功能，生活完全不能自理，卧床不起
ECOG：Eastern Collaborative Oncology Group.	

## 5 检查与分期

### 5.1 术前检查

#### 5.1.1 常规检查

患者需在2周内接受血、尿、大便常规，肝、肾功能，凝血功能，肿瘤标志物，血型检查和感染筛查，心电图、肺功能等检查。

#### 5.1.2 影像检查

患者需在2周内行胸部增强CT检查。为了明确分期，需要进行腹部B超、全身骨扫描、头颅CT或磁共振等检查，或者全身PET-CT检查。

#### 5.1.3 病理检查

经皮肺穿刺活检或者纤维支气管镜活检。对怀疑纵隔淋巴结转移者可行支气管内超声引导下经支气管针吸活检术（endobronchial ultrasound-guided transbronchial needle aspiration, EBUS-TBNA）或纵隔镜检查。

### 5.2 临床分期

见表 3、表 4。

**4:** 第8版NSCLC的TNM分期组合 IASLC Lung Cancer Staging Project: proposed TNM categories and stage groupings for NSCLC

TNM分期			
隐匿癌	T_X_	N_0_	M_0_
0期	Tis	N_0_	M_0_
Ⅰa期	T_1a, b, c_	N_0_	M_0_
Ⅰb期	T_2a_	N_0_	M_0_
Ⅱa期	T_2b_	N_0_	M_0_
Ⅱb期	T_1_	N_1_	M_0_
	T_2_	N_1_	M_0_
	T_3_	N_0_	M_0_
Ⅲa期	T_1, 2_	N_2_	M_0_
	T_3, 4_	N_1_	M_0_
	T_4_	N_0_	M_0_
Ⅲb期	T_3, 4_	N_2_	M_0_
	T_1, 2_	N_3_	M_0_
Ⅲc期	T_3, 4_	N_3_	M_0_
Ⅳa期	任何T	任何N	M_1a_, _b_
Ⅳb期	任何T	任何N	M_1c_
IASLC：国际肺癌研究协会（The International Association for the Study of Lung Cancer）			

## 6 术前准备

### 6.1 制定计划

根据CT或PET-CT描述肿瘤的位置、大小、数目、形状以及与心脏大血管、气管支气管等的关系，确定体位和穿刺通路。

### 6.2 仪器设备

CT、射频消融治疗仪、射频电极、胸穿或胸腔闭式引流包、心电监护仪、吸氧装置、抢救车等相关设备。

### 6.3 药品准备

准备用于麻醉、镇痛、镇咳、止血、扩冠、降压等药物。

### 6.4 患者准备

① 患者及家属（被委托人）签署知情同意书；②术前4 h禁食水；③必要时备皮；④常规建立静脉通道；⑤必要时术前口服镇咳剂；⑥术前教育。

## 7 操作步骤

### 7.1 体位

患者的体位选择要兼顾到穿刺通路的选择。穿刺通路的选择原则是穿刺距离最短，并避开骨骼、肺裂、肺大疱和其他重要结构。而体位选择的原则是患者易于固定和相对舒适。

### 7.2 监测生命体征

消融过程需要监测心率、血压和血氧饱和度，同时要观察患者的呼吸、疼痛、咳嗽、咯血等情况，必要时对症处理。

### 7.3 消毒与麻醉

碘酒、酒精消毒，铺无菌巾；穿刺点处用1%-2%利多卡因局部浸润麻醉，直至胸膜。对于儿童、术中不能配合、预计手术时间长、肿瘤贴近壁层胸膜可能引起剧痛的患者，推荐采用清醒镇静或全身麻醉。麻醉前评估可参照美国麻醉医师协会（American Society of Anesthesiologists, ASA）的分级标准（表 5），≤Ⅲ级的患者方可进行射频消融治疗。

**5:** ASA麻醉风险分级标准 ASA classification of anesthesia risk

分级	标准
Ⅰ级	身体健康，发育营养良好，各器官功能正常
Ⅱ级	除外科疾病外，有轻度合并疾病，功能代偿健全
Ⅲ级	合并疾病较严重，体力活动受限，但尚能应付日常活动
Ⅳ级	合并疾病严重，丧失日常活动能力，经常面临生命威胁
Ⅴ级	无论手术与否，生命难以维持24 h的濒死患者
ASA：美国麻醉医师协会（American Society of Anesthesiologists）.	

### 7.4 定位与穿刺^[43]^

每次CT扫描的范围包括靶肿瘤即可。将射频电极在CT引导下通过穿刺点刺入靶肿瘤。通过CT影像确认射频电极处于预定位置后，进行消融。为确保完全消融靶肿瘤，在安全的前提下，射频电极的覆盖范围应包括靶肿瘤及瘤周0.5 cm-1.0 cm肺组织，即所谓的“消融区”。

### 7.5 消融

根据射频消融治疗仪的类型、射频电极的型号、肿瘤大小及其与周围组织结构的关系设置治疗参数（肺部肿瘤射频消融可以根据不同设备生产商推荐的参数进行适当调整）。消融结束，拔出射频电极前要做针道消融，以减少肿瘤种植和出血。

#### 7.5.1 小肿瘤

直径≤3 cm者，可以单次射频消融。

#### 7.5.2 中肿瘤

直径3 cm-5 cm的肿瘤，单次多点射频消融。

#### 7.5.3 大肿瘤

直径 > 5 cm的肿瘤，单次多点射频消融治疗，必要时辅助放疗或再次射频消融治疗。

#### 7.5.4 特殊部位肿瘤

如邻近心脏大血管、气管支气管、食管、膈肌和胸膜顶病灶，建议使用单电极，穿刺方向尽可能与重要结构平行，并保证距离在0.5 cm以上。

### 7.6 术后扫描

立即进行再次CT全胸腔扫描，评价技术是否成功（肿瘤是否按照消融程序完成治疗和覆盖完全），同时观察是否有并发症的发生。

### 7.7 术后处理

术后平卧2 h-4 h，并监测生命体征。24 h-48 h后拍胸片或CT扫描，观察是否有并发症的发生（如无症状性气胸或胸腔积液）。

## 8 并发症及处理

射频消融是一种相对安全的局部治疗手段，其并发症分级参照美国介入放射学会（Society of Interventional Radiology, SIR）影像引导肿瘤消融国际工作组（International Working Group on Image-Guided Tumor Ablation）的标准^[44]^（表 6）。按照发生时间分为即刻并发症（射频消融后≤24 h）、围手术期并发症（射频消融后24 h-30 d）及迟发并发症（射频消融后 > 30 d）。

**6:** SIR并发症的定义与分级标准 SIR definitions and grading system of complication

并发症分类	定义
副反应	疼痛
	消融后综合症
	无症状胸腔积液
	无后果的邻近结构损伤
轻微	没有不良结果，不需要治疗
	没有不良结果，仅是名义上的治疗，包括仅需要过夜观察
严重	需要住院进行小的治疗 < 48 h
	需要留院进行大的治疗 > 48 h，提升护理级别，延长住院时间
	导致永久不良后遗症
	死亡
SIR：美国介入放射学学会（Society of Interventional Radiology）.	

射频消融治疗肺部肿瘤的并发症分两种：穿刺相关并发症（如肺内出血、血胸、气胸、心包填塞、空气栓塞等）和消融相关并发症（如胸痛、胸膜反应、咳嗽、皮肤灼伤等）^[45]^。肺部肿瘤射频消融的死亡率为0-5.6%^[46]^。在样本量大于100例的文献中，射频消融的死亡率为0-2.2%，严重并发症和轻微并发症发生率分别为3%-24.5%和21.3%-64.9%，其死亡原因有出血、肺炎、肺间质纤维化恶化、肺栓塞、急性心衰、呼吸衰竭等^[47, 48]^。推荐美国国家癌症研究所的通用不良事件术语标准4.03版（Common Terminology Criteria Adverse Events Version 4.03, CTCAE v4.03）^[49]^。

### 8.1 疼痛

推荐CTCAE v4.03报告：0级，没有疼痛；1级，轻度疼痛，不影响功能；2级，中度疼痛，需要止痛药，干扰功能但不干扰日常活动；3级，严重疼痛，需要止痛药，严重影响日常生活活动；4级，伤残性疼痛。

#### 8.1.1 术中疼痛

（1）原因：在局麻条件下手术，一般均有不同程度的疼痛，可能是热传导刺激胸膜神经所致。Okuma等^[50]^单变量和多变量分析研究认为，疼痛的发生与病变距离胸壁在1 cm以内显著相关。（2）治疗：如果疼痛剧烈，需要对胸膜彻底麻醉；或者需要镇痛剂，甚至清醒镇静麻醉；或者降低靶温度到70 ℃，几分钟后，再逐渐升高到靶温度；或者通过三维重建CT图像，观察有无电极针接近胸膜，可以旋转电极针，再消融；或者向胸腔内推电极针，使脏层胸膜离开壁层胸膜，即造成人工气胸^[51, 52]^。

#### 8.1.2 术后疼痛

一般为1级-2级疼痛，可持续数天，也有人持续1周-2周，一般无需特别处理，很少出现中度以上的疼痛，可以用非甾体类药物止痛。

### 8.2 消融后综合征

发生率为6.6%-22.2%（18%）^[46]^。是一过性自限性综合征，表现为低热及其他不适等。（1）原因：肿瘤坏死吸收，其严重程度及持续时间取决于产生坏死的体积以及病人的总体情况，大部分患者症状持续2 d-7 d，消融肿瘤体积较大者则持续2周-3周。（2）治疗：大多数一过性自限性症状，对症支持即可。少数患者需要给予非甾体类药物，必要时可以适量短时应用小剂量糖皮质激素。

### 8.3 气胸

发生率为5%-63%^[46, 50]^。推荐CTCAE v4.03报告：0级，没有气胸；1级，不需要干预；2级，需要放置胸腔闭式引流；3级，需要胸膜固定或手术治疗；4级，威胁生命。Nour-Eldin等^[53]^根据压缩的肺表面到胸膜的距离分为少量气胸（≤2 cm）、中量气胸（2 cm-4 cm）和大量气胸（> 4 cm）。

#### 8.3.1 术中气胸

（1）原因：Hiraki等^[54]^报道发生气胸的危险因素包括男性（肺活量大）、无胸部手术史（没有胸膜粘连）、消融多个肿瘤（多次穿刺）、中下叶病变（肺活动度大）、病变小且深在（难以定位，需反复穿刺）、大肿瘤（多点消融，反复穿刺、集束针、消融时间超过3 h）。Sano等^[55]^研究结果表明高龄、多头伸展型射频电极针和大功率输出是气胸发生的危险因素。Kennedy等^[56]^对10项回顾性研究的1, 916次肺部肿瘤消融结果进行*meta*分析，发现高龄、男性、没有肺部手术史、消融次数多、穿刺深度长等是气胸发生的高危因素。总之气胸的发生率与高龄、合并肺气肿、多次进针、粗针、病变深、穿刺经验有关。上叶肺肿瘤射频消融时，由于肺泡胸膜压力梯度高，气胸的发生率较高^[54]^。（2）治疗：少量气胸可不予处置，中等至大量气胸可胸穿抽气或放置胸腔闭式引流装置^[57]^。文献报道3.3%-38.9%（平均11%）需要放置胸腔闭式引流^[46]^。Hiraki等^[54]^发现，气胸需要放置胸腔闭式引流的高危因素包括：同侧肺部无手术史（*P*=0.002）、使用集束针（*P* < 0.001）、位于上肺叶肿瘤（可能的原因是上叶肺泡胸膜压力梯度高，患者直立时，大量气体持续进入胸腔）（*P* < 0.001）。气胸发生后，是否终止还是继续射频电极的定位操作，取决于抽气后气胸是否有改善、射频电极能否准确定位以及患者的临床症状等。如果经过处理后气胸量减少、患者没有症状，射频电极可以准确定位，建议继续操作；否则可能需要放置胸腔闭式引流，待气胸好转、患者症状改善后再操作。如果患者经过胸腔闭式引流仍然有气体漏出，可以持续负压吸引、行胸膜固定术、气管镜下注入硬化剂、气管内置入阀门等^[58]^。（3）预防：一般来说，穿刺针：①通过叶间裂，气胸的发生率增加3倍；②通过肺大疱；③与胸膜成斜面时气胸的发生率高。为减少气胸的发生，关键在于穿刺技术要熟练，进针速度快和穿刺准确避免多次穿刺十分重要。拔出射频电极针后患者取穿刺侧在下卧位，吸氧可降低气胸发生率。

#### 8.3.2 迟发性气胸

发生率约10%^[59, 60]^。一般认为消融后72 h发生的气胸称为迟发性气胸，处理同前。有研究者^[53]^提出无同侧肺部手术史、病灶深在和射频消融后靶肿瘤的GGO紧邻胸膜是发生迟发性气胸或复发性气胸的高危因素。针道消融后胸膜周围组织干燥，不利于弹性回缩封闭针孔，可能发生支气管胸膜瘘，甚至发展成张力性气胸，需要特别关注。

#### 8.3.3 皮下气肿

发生率0.2%^[61]^。在射频消融过程中，发生气胸时，如果胸膜腔粘连，气体沿穿刺针道进入皮下而形成皮下气肿。如果气胸量不大或者经过处理，皮下气肿可逐渐吸收。

### 8.4 胸腔积液

消融后经常可以见到少量胸腔积液，发生率1.3%-60%（13.4%）^[46]^。推荐CTCAE v4.03报告：0级，没有胸腔积液；1级，无症状和不需要干预；2级，有症状，需要利尿；3级，有症状，需要吸氧或胸腔穿刺；4级，威胁生命（需要气管插管）。（1）原因：与消融过程中高温胸膜受刺激有关。导致胸腔积液发生的危险因素有：合并慢阻肺、病灶大、一次消融多个病灶、病灶靠近胸膜（< 10 mm）、消融时间长等^[54]^。（2）治疗：一般观察或保守处理即可。如果出现中到大量胸腔积液，需要行穿刺抽吸或胸腔闭式引流，需要胸腔引流者低于10%。（3）预防：消融时尽量远离胸膜。

### 8.5 出血

术中咯血发生率3.3%-18.2%（11.1%）^[46]^，大咯血的发生率极低。肺内出血发生率0-11%（7.1%）与咯血和术后血痰并不一致。血胸发生率1.9%-16.7%（4.3%）。（1）原因：没有发现特殊的高危因素^[50]^。但也有人认为与病灶小、穿刺路径长、合并慢阻肺、肺动脉高压有关。（2）治疗：术中出现咯血后立即消融有利于止血。肺内出血可自动吸收。术后血痰多具有自限性，可持续3 d-5 d。如果术中发现少量胸腔积液，可以密切观察，保守治疗；如果出现中到大量胸腔积液，说明有活动出血，需要行穿刺抽吸或胸腔闭式引流，文献报道约10%左右需要胸腔闭式引流，同时应用止血药物。血胸保守治疗无效者，可行介入栓塞治疗或剖胸探查。（3）预防：由于消融本身可以使血液凝固，随着消融的进行出血会逐渐停止，故在消融过程中大出血的发生率并不高。穿刺时避开血管走行区或者不张的肺组织等。术前要注意血小板计数、凝血功能和抗凝药的应用等。

### 8.6 咳嗽

推荐CTCAE v4.03报告：0级，没有咳嗽；1级，不需要干预可以缓解；2级，需要止咳药缓解；3级，严重咳嗽或痉挛性咳嗽，对治疗无效。（1）原因：术中剧烈咳嗽可能与病灶局部温度升高刺激肺泡、支气管内膜或胸膜所致。术后咳嗽是消融后局部肿瘤组织坏死及其周围肺组织热损伤引起的炎症反应所致。（2）治疗：口服镇咳剂或经过射频电极注水孔注入利多卡因即可缓解，部分患者可能只有在消融结束后咳嗽才停止。术后咳嗽可适当给予止咳化痰药。（3）预防：消融前半小时含服可待因可减轻咳嗽反应。

### 8.7 胸膜反应

（1）原因：①消融过程中刺激了支配壁层胸膜的迷走神经，兴奋的迷走神经可使心率减慢、甚至心跳停止。②局部麻醉不充分；部分患者对疾病不了解，对治疗手段恐惧，甚至处于高度紧张状态；病变距离胸膜在1 cm以内。（2）治疗：针对这类患者，建议暂停消融，局部充分麻醉，并适当应用阿托品、镇静剂等药物。（3）预防：术前沟通，患者精神放松，或者彻底麻醉附近胸膜。

### 8.8 肺部炎症

肺炎发生率6%-12%（9.5%）、肺脓肿为1.9%-6.6%（6.4%）^[46]^。更少见的是闭塞性细支气管炎（bronchiolitis obliterans organizing pneumonia, BOOP），它是一种射频消融术后的反应性肺炎，可能是肉芽组织增生引起的支气管狭窄和阻塞导致远端阻塞性肺炎^[62]^。发生率0.4%（3/840），表现为非特异性症状（如发热、咳嗽、咳痰、呼吸困难），CT表现为肺周围结节样或GGO，或斑片状含气阴影，对抗生素无效，但是对类固醇激素冲击疗法有效。（1）原因：发生的高危因素有年龄 > 70岁、免疫力低下或放疗后的老年患者，合并慢阻肺、间质性肺炎和糖尿病，肿瘤 > 4 cm。（2）治疗：若术后5 d体温仍 > 38.5 ℃，首先考虑肺部感染，应摄胸部平片或行胸部CT扫描（推荐）予以确认，并根据痰液、血液或脓液培养结果调整抗生素；如胸片或胸部CT扫描提示肺内/胸腔脓肿应置管引流。感染的最坏结果是可能发展成为急性呼吸窘迫综合征（acute respiratory distress syndrome, ARDS）甚至死亡。（3）预防：术前充分评价肺功能，并对肺部合并疾病进行处理。

### 8.9 少见并发症

其他潜在致命的并发症包括支气管胸膜瘘、空气栓塞、肺动脉假性动脉瘤和心包填塞。其他严重并发症包括邻近神经损伤（如臂丛、肋间、膈、喉返等神经对热敏感）、针道种植、肺脓肿、皮肤灼伤等^[63]^。

## 9 随访及疗效评估

### 9.1 随访

一般评价包括患者的症状体征、肿瘤标志物、T细胞亚群、体力状态评分、肺功能、生活质量等。局部疗效评价常选择胸部CT检查，有条件者可选择PET-CT检查，主要用于评价靶肿瘤是否完全消融，有无局部进展、新发病灶等。术后4周-6周复查胸部增强CT，并以此为基线进行评价，术后2年内每3个月复查胸部CT，2年后每6个月复查一次。PET-CT可以在消融后3个月或6个月第一次复查，以后每6个月复查一次，2年后每年复查一次。PET-CT检查判断疗效更准确，并有助于确定有无肺外转移。远期疗效评价包括无疾病进展生存（progression free survival, PFS）即治疗开始至出现影像学进展或者死亡的时间间隔；总生存率（overall survival, OS）和肿瘤特异生存率（cancer specific survival, CSS）等。技术效率和安全性评价至少随访6个月；近期疗效评价至少随访1年；中期疗效评价至少随访3年；长期疗效评价至少随访5年。

### 9.2 消融区影像学改变^[64, 65]^

#### 9.2.1 CT改变

##### 9.2.1.1 早期改变（1周内）

消融区增大，病灶内蜂窝状低密度改变，周边包绕宽度大于5 mm的环周或部分GGO是治疗成功的表现。但是，单纯用GGO衡量有可能高估消融效果，该区域病理学检查可见“鬼影”细胞，可能是由于热消融后肿瘤突然凝固性坏死和微循环破坏，阻止酶从细胞内溶酶体释放，以及炎性细胞浸润，延迟细胞自溶所致，需要用还原型烟酰胺腺嘌呤二核苷酸（NADH）离体活体染色及其他特殊染色进行鉴别。

##### 9.2.1.2 中期改变（1周-3个月内）

消融区增大，其周边由于炎症吸收可能出现环周清晰锐利的强化。

##### 9.2.1.3 后期改变（3个月后）

与基线相比，消融区在3个月后保持稳定或稍大，6个月后大小稳定或逐渐缩小，并可有多种演变模式（如纤维化、空洞形成、结节、肺不张、消失等），或组合出现。

#### 9.2.2 PET-CT改变

用标准摄取比（standard uptake value, SUV）描述。

### 9.3

局部疗效评估：推荐使用改良的实体瘤疗效评价标准（modifield response evaluation criteria in solid tumors, mRECIST）^[66]^。见表 7。

**7:** 改良实体瘤疗效评价标准 Modified RECIST criteria

效果	CT（大小）	CT（密度）	PET或PET-CT
完全消融	缩小或不变	无强化区	无高代谢区
不完全消融	不变或增大	强化区无变化	高代谢区无变化
局部进展	新增大于10 mm	新增强化区	新增高代谢区
RECIST：实体瘤疗效评价标准（Response Evaluation Criteria in Solid Tumors）.			

#### 9.3.1 完全消融

CT检查提示出现下列表现任何一项，如靶肿瘤消失，无强化的空洞、实性结节、肺不张和纤维化等。或者PET-CT检查提示靶肿瘤无核素浓聚或SUV值正常。

#### 9.3.2 不完全消融

CT检查提示靶肿瘤空洞形成不完全，有部分实性或液性成分，且强化CT扫描有强化；靶肿瘤部分纤维化仍存有部分实性成分，且实性部分强化CT扫描有强化；靶肿瘤呈实性结节，大小无变化或增大，且伴强化CT扫描有强化。PET-CT检查提示靶肿瘤消融后仍有核素浓聚或SUV值仍高于正常。

#### 9.3.3 肿瘤局部进展

CT检查提示靶肿瘤完全消融后，瘤周又出现散在、结节状、不规则偏心强化；PET-CT检查提示消融后靶肿瘤无核素浓聚或SUV值正常后，又出现核素浓聚或SUV值高于正常。对局部肿瘤进展的患者需要进行二次消融或其他治疗。

#### 9.3.4 首次和重复技术效率

首次技术效率定义为在随访期间内首次消融治疗成功的比率。重复技术效率指肿瘤局部进展后再次消融治疗成功的比率。

### 9.4 远期疗效评估

消融后随访1年-5年，记录患者生存状况。

## 10 综合治疗

射频消融联合其他方法进行治疗是目前肺部肿瘤研究的重要内容之一，包括射频消融与外科^[39]^、放疗^[67, 68]^、化疗^[69]^和分子靶向药物^[70-72]^等的联合，可以提高肿瘤的局部控制率，延长患者的生存。

## 11 总结

最近研究表明：射频消融治疗不能手术的早期NSCLC（肿瘤直径≤3 cm）的1年、3年和5年的生存率分别达到90%、70%和50%，且死亡率小于2%^[73]^。这些临床证据让我们相信这一技术可能会成为继手术、放疗、化疗之后的第四大治疗手段。但是还需要前瞻性多中心随机临床研究加以证实^[74]^，并与其他治疗手段如手术^[75-78]^、消融^[79]^和立体定向放射治疗^[80-83]^进行比较；再者，射频消融作为一种局部微创消融治疗手段，需要联合其他治疗手段提高疗效，比如放疗、全身治疗（根据基因检测结果，选择用化疗或者分子靶向药物治疗）等。

总之，从临床实践的角度看，有关射频消融技术治疗肺部肿瘤的有效性和安全性都已得到临床验证，但是不同单位开展的方法并不一致，希望通过本共识达到规范化推广的目的。本共识虽然借鉴了许多国际指南和国内外的最新进展，经过多次认真讨论和反复修改，仍难免存在不足和局限性，因此，需要在以后的临床实践中不断补充，动态完善，制定出符合我国国情的射频消融技术临床指南。
